# Neuroimaging reveals distinct brain glucose metabolism patterns associated with morphine consumption in Lewis and Fischer 344 rat strains

**DOI:** 10.1038/s41598-022-08698-9

**Published:** 2022-03-17

**Authors:** Mª Luisa Soto-Montenegro, Verónica García-Vázquez, Nicolás Lamanna-Rama, Gonzalo López-Montoya, Manuel Desco, Emilio Ambrosio

**Affiliations:** 1 Instituto de Investigación Sanitaria Gregorio Marañón, Madrid, Spain; 2 CIBER de Salud Mental (CIBERSAM), Madrid, Spain; 3 Departamento de Bioingeniería E Ingeniería Aeroespacial, Universidad Carlos III de Madrid, Madrid, Spain; 4 Departamento de Psicobiología, Facultad de Psicología, Universidad de Educación Nacional a Distancia (UNED), Madrid, Spain; 5 Centro Nacional de Investigaciones Cardiovasculares, CNIC, Madrid, Spain; 6 Laboratorio de Imagen, Medicina Experimental, Hospital General Universitario Gregorio Marañón, Dr. Esquerdo, 46, 28007 Madrid, Spain

**Keywords:** Neuroscience, Diseases, Neurology

## Abstract

Vulnerability to addiction may be given by the individual's risk of developing an addiction during their lifetime. A challenge in the neurobiology of drug addiction is understanding why some people become addicted to drugs. Here, we used positron emission tomography (PET) and statistical parametric mapping (SPM) to evaluate changes in brain glucose metabolism in response to chronic morphine self-administration (MSA) in two rat strains with different vulnerability to drug abuse, Lewis (LEW) and Fischer 344 (F344). Four groups of animals were trained to self-administer morphine or saline for 15 days. 2-deoxy-2-[^18^F]-fluoro-d-glucose (FDG)-PET studies were performed on the last day of MSA (acquisition phase) and after 15 days of withdrawal. PET data were analyzed using SPM12. LEW-animals self-administered more morphine injections per session than F344-animals. We found significant brain metabolic differences between LEW and F344 strains in the cortex, hypothalamus, brainstem, and cerebellum. In addition, the different brain metabolic patterns observed after the MSA study between these rat strains indicate differences in the efficiency of neural substrates to translate the drug effects, which could explain the differences in predisposition to morphine abuse between one individual and another. These findings have important implications for the use of these rat strains in translational morphine and opiate research.

## Introduction

Addiction vulnerability is the individual's risk of developing an addiction during their lifetime. Clinical and preclinical studies have shown that among the biological factors involved in this disorder, genetic variability plays an important role in humans^1–4^, accounting for at least 40–60% of the variation in vulnerability to drug dependence^5^. In this regard, the use of inbred rats has proven to be a valuable tool for identifying differences in vulnerability to drug addiction. The Lewis (LEW) and Fischer 344 (F344) inbred rat strains have been the most widely used in modeling genetic vulnerability to drugs. LEW and F344 rats differ with respect to drug self-administration. Consequently, LEW rats more readily self-administer drugs, such as alcohol, opiates, and cocaine, than F344 rats^6–13^. At the neurochemical level, F344 rats have high basal levels of tyrosine hydroxylase (TH) protein, proenkephalin, dopamine transporter, glutamate, and μ-opioid receptors in the nucleus accumbens (NAcc)^14,15^. F344 rats also have high levels of μ-opioid receptors in the striatum, lateral globus pallidus, basolateral and lateral amygdaloid nucleus, periaqueductal gray matter (PAG), substantia nigra, and locus coeruleus measured by autoradiography^11^. LEW rats have lower active dopaminergic neurons in the ventral tegmental area (VTA)^16^ but greater increases in extracellular dopamine in ventral striatum and lower levels of dopamine metabolites than F344 rats^17^. In addition, LEW rats have high basal levels of TH protein and dopamine D1 and NMDA receptors in the VTA^12,18–20^, as well as greater activity of the μ-opioid receptor in the nucleus accumbens, septal nuclei, thalamus, VTA, raphe nuclei, and locus coeruleus^11^; but lower basal levels of glutamate and GABA in the NAcc^21^. Furthermore, both strains show differences in the reactivity of the hypothalamic-pituitary-adrenocortical axis, such as reduced corticosterone responses to stress and morphine in LEW-animals among others^22,23^. Recently, genetic factors have been involved in the control of the vulnerability to drugs of abuse, with differences in the transcription of NGFI-B and Nor1 in the caudate-putamen, involved in the control of behaviors^24^, or signalling pathway of mTOR (Raptor and Eif4ebp2 expression) that has an important role in the long-lasting neuroadaptations that occur on the progression of addictive behaviour in the amygdala^13^.

In line with these observations, functional neuroimaging techniques, such as positron emission tomography (PET), single-photon emission computed tomography, functional magnetic resonance imaging (fMRI), and electro-encephalography, have been widely used to demonstrate the emotional and cognitive-behavioral components of addiction^25,26^, as well as to study the neurotoxic effects of drugs in the brain^27–34^. In this respect, PET with 2-deoxy-2-[^18^F]-fluoro-d-glucose (FDG) has traditionally been the most commonly used technique for direct quantification of regional brain glucose metabolism in clinical and preclinical studies^35–40^. It constitutes an invaluable tool for investigating the in vivo changes in brain metabolism as a result of pharmacological manipulation. Given that the LEW and F344 strains show differential behaviours that model addiction and therefore different vulnerability to drugs, our working hypothesis is that the metabolism of morphine in reward areas of the brain would be higher in the LEW strain than in the F344 strain. As far as we know, this is the first study to examine the effects of morphine self-administration on glucose metabolism using in vivo FDG-PET and statistical parametric mapping (SPM) in two rat strains with different vulnerability to drug abuse.

## Methods

### Animals

Thirteen male F344 and fourteen LEW inbred rats (250–300 g) were obtained from the animal facility of Universidad Nacional de Educación a Distancia (UNED). Animals were housed individually when they reached post-natal day 75 (PND75) to leave sufficient time between individual housing and brain metabolic studies and thus avoid any non-specific effects of isolation stress on the metabolic measurements. All animal procedures were conducted in conformity with Directive 2010/63/EU of the European Parliament and of the Council, the ARRIVE guidelines, and approved by the Ethics Committee for Animal Experimentation of UNED and Hospital Gregorio Marañón (number ES280790000087).

### Drug administration and experimental protocol

**Figure 1:**
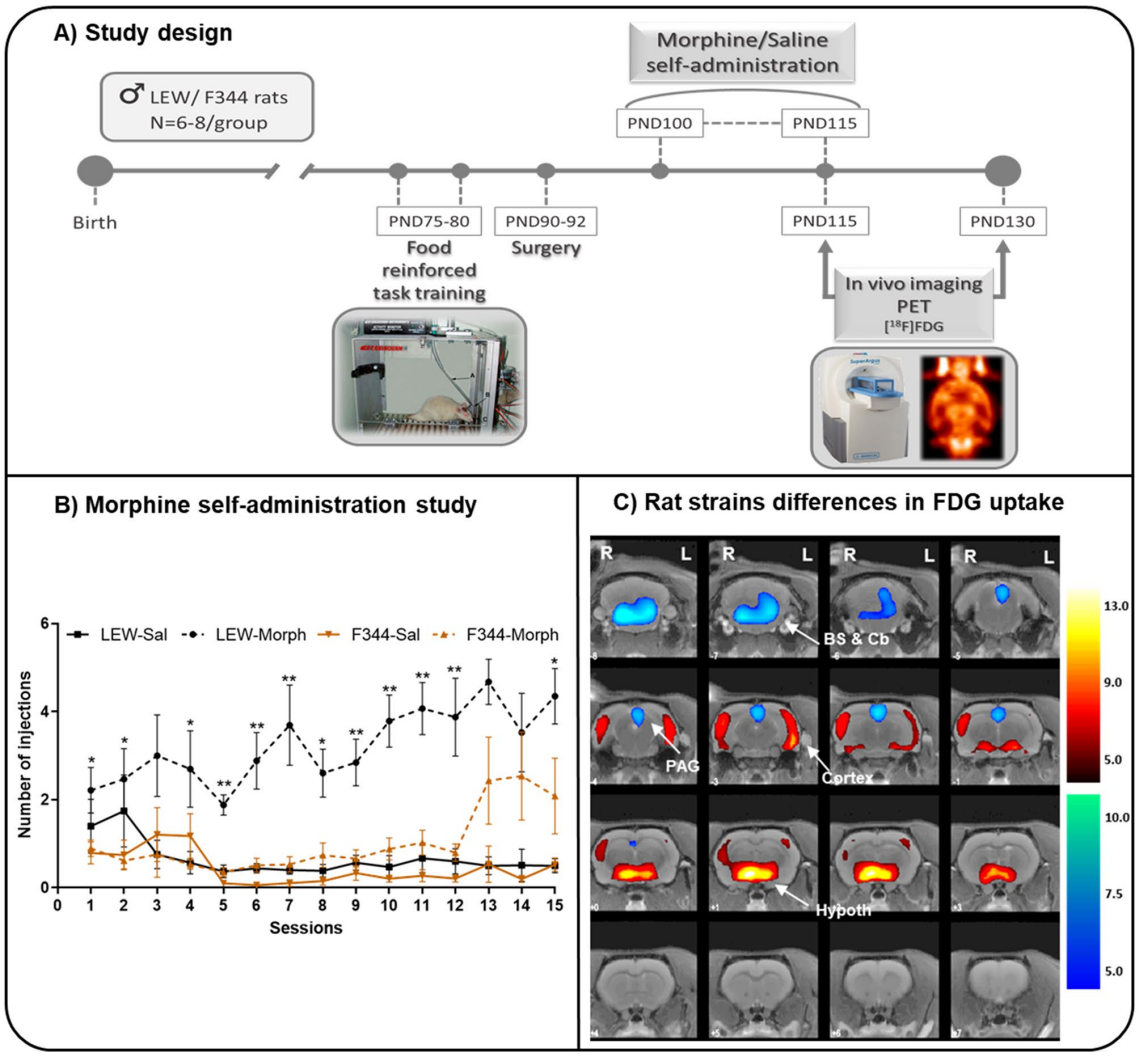
Study design, behavioral study and differences in brain glucose metabolism between rat strains. **(A)** Representative diagram of the chronology of the experimental procedures performed during the study according to the age of the animals. Abbrev.: ^18^FDG, [18F]-Fluorodeoxyglucose; PET, positron emission tomography. **(B)** MSA study in LEW and F344 rats. Morphine (1 mg/kg) or saline self-administration in adult LEW and F344 rats under an FR1 schedule of reinforcement. The values are expressed as the mean ± SEM: LEW-saline (n = 6), LEW-morphine (n = 8), F344-saline (n = 6), and F344-morphine (n = 7). The number of morphine injections per session was greater for LEW animals than for F344 animals. 3-way ANOVA followed by Bonferroni’s multiple comparisons test [*p < 0.05 and **p < 0.01 vs saline animals]. (**C)** PET results in T-maps overlaid on aT2-MRI reference showing increased FDG uptake (hot colors) or decreased FDG uptake (cold colors) in Saline-LEW animals compared with Saline-F344 animals in the first scan (PND 115). Similar results were obtained for the second scan (PND 130) (data not shown). Saline-LEW animals showed higher FDG uptake in the hypothalamus and the cerebral cortex and lower FDG uptake in the brainstem, cerebellum and PAG than Saline-F344 animals. Statistics corrected for multiple comparisons (FWE, p < 0.05). *Region of interest (BS: brainstem, Cb: Cerebellum, Hypoth: hypothalamus, PAG: periaqueductal gray matter). Side: left (L) and right (R). k: cluster size, T: Student t. FDG uptake: increase (↑) and decrease (↓). p: p value (unc: uncorrected, FWE: family-wise error).*

Figure 1A shows the drug treatment and the design of the study. Twelve operant chambers (Coulbourn Instruments, Allentown, PA, USA) were used for the operant food-reinforced behaviour and morphine self-administration studies. A lever designed to register a response to 3.0 g of force was placed on the front wall of the chamber^11^. Food and morphine operant data were acquired and stored on IBM computers (Med Associates, PA, USA).

Saline or morphine sulphate self-administration (1 mg/kg/injection in 12 h daily sessions) was studied with a fixed-ratio 1 (FR1) schedule of reinforcement for 15 days^13^.

Animals were divided into four groups based on the factors studied: rat strain (LEW and F344) and drug treatment (morphine and saline). Sample size of the groups were: 1) LEW-saline (n = 6); 2) LEW-morphine (n = 8); 3) F344-saline (n = 6); and 4) F344-morphine (n = 7).

#### Fixed-ratio1 food-reinforced behavior

This experimental procedure was performed at PND75. The FR1 schedule of food reinforcement is a commonly used behavioral procedure to determine whether a drug is reinforcing or not^41^. To do this, animals are trained and learn the association between behavior (pressing the lever) and response (pellet reward). Before training begins, animals were food deprived to 95%-90% of their free-feeding weight and submitted to an FR1 schedule of food reinforcement in which a single press of the lever turned on a light stimulus above the lever that signalled pellet delivery and reward availability (45 mg; Noyes Pellets, USA). Each pellet delivery was followed by a 30-s timeout period in which responses had no scheduled consequence (FR1: TO 30 s). This food-reinforced behavior was acquired over 5 days (30 min each day). After this phase, the animals had ad libitum access to food and were able to recover their free-feeding weight.

#### Morphine self-administration (MSA)

When the response rate was sufficient (more than 50 responses per session) and stable (less than 10% variation across three consecutive sessions), animals were operated with an intravenous catheter in the right jugular vein. Polyvinylchloride tubing (0.064 i.d.) was implanted approximately at the level of the atrium under anesthesia with ketamine (40 mg/kg, i.p.) and diazepam (10 mg/kg, i.p.). The catheter was tunnelled and located in the midscapular region; it then passed through a spring tether system (Alice King, Chatham, CA, USA) that was mounted on the skull of the rat with dental cement. Animals were given at least five days to recover from the surgery, and the catheters were flushed daily with 0.5 ml of an antibiotic solution (gentamicin 40 mg/ml) dissolved in heparinized saline to prevent infection and to maintain catheter patency.

The drug-reinforced behaviour study was performed during the dark cycle since rodents are nocturnal animals, coinciding with their period of greatest activity. Animals did not have free access to food during MSA protocol. It involved two phases: 1) The acquisition phase, which started at PND100 and consisted of sessions lasting 12 h per day (starting at 8:00 pm) with morphine sulphate (1 mg/kg, dissolved in saline [0.9% NaCl] solution) or saline alone under an FR1 schedule of reinforcement for 15 consecutive days; and 2) The withdrawal phase, where the drugs were discontinued for 15 consecutive days. During acquisition sessions, one active lever press resulted in morphine/saline infusion delivered over 10 s and followed by a 10-s time-out. A light cue located above the active lever indicated the availability of the drug; this was turned off only during drug delivery, time out, and at the end of each session. A limit of 50 infusions per session was set in order to avoid overdosing.

### FDG-PET imaging studies

PET images were acquired on two separate days: the first session was at the end of the MSA phase (− acquisition phase −) and the second session was at the end of the withdrawal phase.

Imaging was performed using a dedicated small animal PET scanner (rPET, SUINSA Medical Systems, Madrid). 2-deoxy-2-[^18^F]fluoro-d-glucose (FDG: 73.63 ± 8.88 Mbq) was administered as a regular injection through the catheter inside the jugular vein, and, after an uptake period of 35 min, the animals were imaged for 60 min under isoflurane anesthesia (5% for induction and 1–1.5% for maintenance in 100% O_2_).

Tomographic images were reconstructed with a 3D filtered back projection (3D-FBP) algorithm^42^ using a 12th-order Butterworth filter at a 35% Nyquist frequency cut-off. The FOV of our system was 68 mm transaxial and 47 mm axial. The trans-axial and axial resolutions of the PET scanner were 1.65 mm and 1.9 mm full width at half-maximum (FWHM), respectively. The voxel size of the reconstructed images was 0.81 × 0.81 × 0.81 mm^3^, the energy window was 400–700 keV, and decay, alignment, normalization, and deadtime corrections were applied. The decay correction was based on the theoretical half-life of the radiosotope used. The normalization correction was based on the acquisition of an uniform and homogeneous phantom consisting of a cylinder of ^68^Ge of the appropriate size for the FOV system And finally, the alignment and deadtime corrections were based on periodic calibrations of the equipment according to the manufacturer's instructions^43^.

### Magnetic resonance study

An MRI study of one animal at PND100 was acquired with a 7-Tesla Biospec 70/20 scanner (Bruker, Ettlingen, Germany). A 1H linear volume coil from Bruker was used for an homogeneous excitation of the sample and a 1H receive-only 2 × 2 rat brain array surface coil from Bruker was used for the reception of the signal. The animal was anesthetized with sevoflurane (4.5% for induction and 2.5% for maintenance in 100% O_2_) and placed on a stereotactic device to prevent movement during the acquisition. A T2-weighted spin echo sequence was acquired, with TR = 4062 ms, TE = 33 ms, flip angle = 90°, RARE factor = 8. FOV = 3.7 × 3.7 cm, matrix size = 256 × 256, slice thickness = 0.8 mm (37 slices). The inhomogeneity of magnetic field caused by the surface antenna was corrected.

This MRI study was only used as an anatomical template in order to display the results of the statistical analysis, since PET imaging has a lower anatomical resolution. This MRI template corresponds to a male Wistar rat with the same age and weight as the F344 and LEW animals, with no anatomical differences between then since they are inbred rats of the Wistar strain.

### Data analysis

#### Morphine self-administration

The statistical analysis of behavioral data consisted in a repeated-measures three-way ANOVA including the strain (LEW vs F344), treatment (morphine vs saline), and repetition time as factors. The average number of self-administered injections was also determined and analyzed using two-way ANOVA followed by a post hoc Bonferroni correction.

#### PET: statistical parametric mapping analysis

PET image post-processing and intensity normalization were performed following protocols previously described by our group^44,45^. Briefly, the reconstructed images were spatially registered using rigid transformations with an automatic algorithm based on mutual information^46^. All PET data were smoothed with a 2-mm FWHM isotropic Gaussian kernel. A brain mask was manually segmented onto the MRI template and applied to the PET studies to remove extracerebral voxels and to ensure that only voxels mapping brain tissue were included in the analysis. Image intensity was normalized to the brain average value (100%).

The statistical analysis was performed using SPM12 (http://www.fil.ion.ucl.ac.uk/spm/software/spm12/) and consisted in the analysis of variance (ANOVA) of three fixed factors with repeated measures on one of them: strain (inter-subject factor; levels: LEW, F344), condition (inter-subject factor; levels: saline, morphine), and time (within-subject factor, levels: at the end of the MSA phase, at the end of the withdrawal phase). Results were considered significant at a threshold of *p* < 0.01, uncorrected at the voxel level, but cluster-based–corrected by Family-Wise Error (FWE) in order to avoid a type II error. Only strain differences were significant for multiple comparisons using the family-wise error (FWE) rate with a significance level of *p* < 0.05. A 10-voxel clustering (spatial-extent) threshold was also applied to reduce the possibility of a type I error; therefore, significant regions smaller than ten adjacent activated voxels were not admitted.

### Ethical approval

All applicable international, national, and/or institutional guidelines for the care and use of animals were followed.

## Results

### Morphine self-administration

The three-way ANOVA revealed a statistically significant effect of the strain (*p* < 0.01), treatment (*p* < 0.001), and time (*p* < 0.001). We also found an interaction for strain × treatment (*p* < 0.001) and time × treatment (*p* < 0.001). Thus, the number of morphine injections per session was greater for LEW animals than for F344 animals (*p* < 0.01) (Fig. 1B). In addition, the average number of self-administered injections over the 15 sessions was greater in the LEW-morphine (48.58 ± 6.71) group than in the LEW-saline (9.90 ± 3.32) and F344 rats (morphine: 15.36 ± 4.40; saline: 6.66 ± 1.79). From almost the beginning, LEW-morphine animals showed an increasing learning curve that was maintained asymptotically the rest of sessions. In contrast, F344 animals started an increasing learning curve in the last 3 sessions.

### PET: Statistical Parametric Mapping analysis

Table 1 shows the differences in brain glucose metabolism between rat strains, and the brain metabolic changes after the MSA study.

**Table 1:** (1) Brain metabolic changes in F344 and LEW animals after MSA study. The comparison shows differences in glucose brain metabolism between morphine-treated animals and saline-treated animals, in the acquisition phase (1.A) and the withdrawal phase (1.B). (1.A) F344-morphine animals showed higher FDG uptake in the cortical area and lower FDG uptake in the motor and piriform cortex than the F344-saline animals. LEW-morphine animals showed lower FDG uptake in the somatosensorial and cingulate cortex and the thalamus and higher FDG uptake in the cerebellum than the LEW-saline animals. (B) F344-morphine animals showed higher FDG uptake in the left cortex and cerebellum and lower FDG uptake in the restrosplenial and motor cortices than the F344-saline animals. LEW-morphine animals showed higher FDG uptake in the cerebellum and piriform cortex and lower FDG uptake in the cortex, thalamus, hippocampus, and caudate putamen than the LEW-saline animals. (2) Differences in brain glucose metabolism between LEW-Sal animals and F344-Sal animals. Saline-LEW animals showed higher FDG uptake in the hypothalamus and the cerebral cortex and lower FDG uptake in the brainstem, cerebellum and PAG than Saline-F344 animals. ROI: Region of interest. Side: left (L) and right (R). k: cluster size, T: Student t. FDG uptake: increase (↑) and decrease (↓). p: p value (unc: uncorrected, FWE: family-wise error). *Abbrev.: BS: brainstem; C: cortex; Cb: cerebellum; CC: cingulate cortex; CP: caudate-putamen; Ent C: entorhinal cortex; Hipp: hippocampus; Hypoth: hypothalamus; PAG: periaqueductal gray matter; MC: motor cortex; Pir C: piriform cortex; RSC: retrosplenial cortex; SSC: somatosensorial cortex; Th: thalamus].*

		1. Brain metabolic changes after the MSA study														
		(A) ACQUISITION PHASE							(B) WITHDRAWAL PHASE							
		ROI	Side	t-value	p_unc_ peak	p_FWE_ peak	P_FWE_ cluster	K	ROI	Side	t-value	p_unc_ peak	p_FDR_ peak	p_FDR_ cluster	K	
F344	**↑**	Cortex	R	4.42	< 0.001	0.128	0.053	129	Cortex	L	4.28	< 0.001	0.160	0.197	67	
			L	13.08		0.145	0.248	57	Cb	–	4.05		0.230	0.254	56	
	↓	MC	–	4.66	< 0.001	0.085	0.216	63	RSC-MC	–	5.00	< 0.001	0.046	0.055	127	
		Pir C	R	4.03		0.237	0.700	13								
			L	3.62	0.001	0.421	0.314	47								
LEW	**↑**	Cb	–	3.57	0.001	0.452	0.272	53	Cb		3.54	0.001	0.467	0.079	109	
									Pir C	R	5.32	< 0.001	0.026	0.361	41	
										L	3.70	0.001	0.380	0.505	27	
	↓	SSC-Th-CC	L	4.37	< 0.001	0.137	0.076	111	C-Th-Hipp-CP-CC	R	4.63	< 0.001	0.089	< 0.001	< 0.001	
										L	5.09		0.009			

		2. Differences in brain glucose metabolism between rat strains														
		ROI	Side	t-value	p_unc_ peak	p_FWE_ peak	P_FWE_ cluster	K		ROI	Side	t-value	p_unc_ peak	p_FWE_ peak	P_FWE_ cluster	K
	↑	Hypoth	–	13.08	< 0.001	< 0.001	< 0.001	420	**↓**	BS & Cb	–	9.72	< 0.001	< 0.001	< 0.001	257
		Cortex	R													
			L	9.72	< 0.001	< 0.001	< 0.001			PAG	–	7.49	< 0.001	< 0.001	< 0.001	

SPM analysis revealed a significant effect of the rat strain with higher metabolism in the hypothalamus and the cerebral cortex and lower FDG uptake in the brainstem (locus coeruleus) and PAG in the Saline-LEW animals than in the Saline-F344 animals (Fig. 1C). Similar results were obtained for the first (PND 115) and second scan (PND 130) (data not shown).

**Figure 2:**
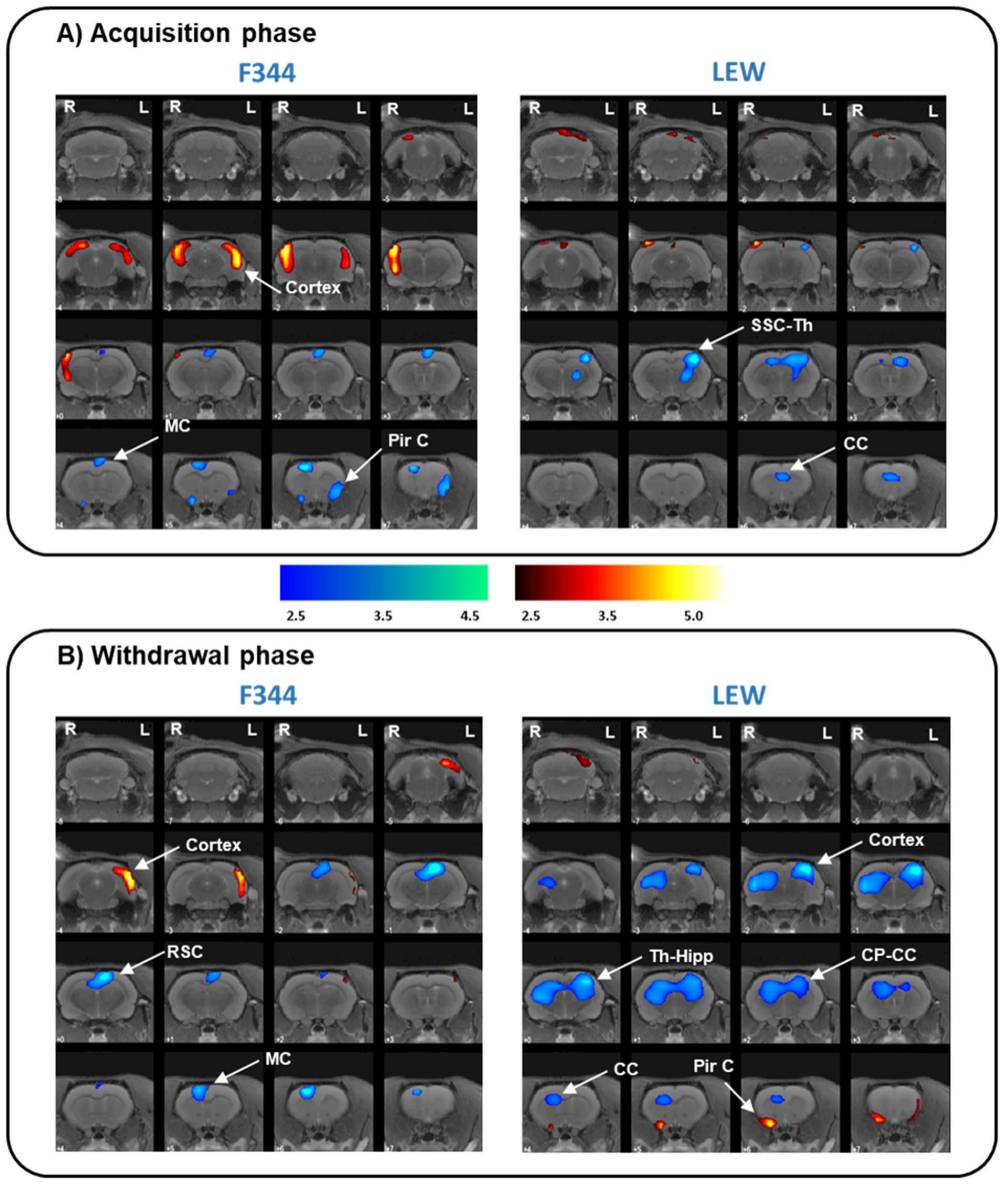
Brain glucose metabolism patterns associated with morphine consumption in F344 and LEW rat strains. PET results in T-maps overlaid on a T2-MRI reference showing increased FDG uptake (hot colors) or decreased FDG uptake (cold colors). PET results in the F344-morphine (left) and LEW-morphine (right) animals compared to the Sal-F344 and Sal-LEW animals respectively, in the acquisition phase (A) and the withdrawal phase (B). (A) F344-morphine animals showed higher FDG uptake in the cortical area and lower FDG uptake in the motor and piriform cortex than the F344-saline animals. LEW-morphine animals showed lower FDG uptake in the somatosensorial and cingulate cortex and thalamus and higher FDG uptake in the cerebellum than the LEW-saline animals. (B) F344-morphine animals showed higher FDG uptake in the left cortex and lower FDG uptake in the restrosplenial and motor cortices than the F344-saline animals. LEW-morphine animals showed higher FDG uptake in the cerebellum and piriform cortex and lower FDG uptake in the cortex, thalamus, hippocampus, and caudate putamen than the LEW-saline animals. LEW-saline (n = 5), LEW-morphine (n = 6), F344-saline (n = 7), and F344-morphine (n = 6). Threshold for statistical significance of p < 0.01. *Region of interest (C: cortex, CC: cingulate cortex, Cb: Cerebellum, CP: caudate putamen, Hipp: hippocampus, MC: motor cortex, PirC: piriform cortex, RSC: retrosplenial cortex, Sept: septum, SSC: somatosensorial cortex; Th: thalamus). Side: left (L) and right (R). k: cluster size, T: Student t. FDG uptake: increase (↑) and decrease (↓). p: p value (unc: uncorrected, FWE: family-wise error).*

During the drug acquisition phase, F344-morphine animals showed higher FDG uptake in the cortical area and lower FDG uptake in the motor and piriform cortex than the F344-saline animals (Fig. 2A). LEW-morphine animals showed lower metabolism in the somatosensorial and cingulate cortex and the thalamus and higher FDG uptake in the cerebellum than the LEW-saline animals (Fig. 2A).

During drug withdrawal, F344-morphine animals showed higher metabolism in the left cortex and lower FDG uptake in the restrosplenial and motor cortices than the F344-saline animals (Fig. 2B). LEW-morphine animals showed higher metabolism in the cerebellum and piriform cortex and lower FDG uptake in the cortex, thalamus, hippocampus, and caudate putamen than the LEW-saline animals (Fig. 2B).

## Discussion

To our knowledge, this is the first report to show that brain glucose metabolism differs from strain to strain, and that this finding may account, among others, for the differences in morphine self-administration (MSA) between LEW and F344 animals.

One of the core traits shown by rat strains with differential vulnerability to drug addiction is the capacity to self-administer more drug than other rat strains^1,4,47,48^. According to this, drug intake is very high in some *vulnerable* animals and very low in other *resistant* ones^48^. In this regard, LEW rats are more likely to transition to addiction and are more likely to relapse than F344 rats^9^.

In this study, we found that LEW rats self-administered more morphine injections per session than F344 rats, as previously reported by others and also by our group^9–11^. Furthermore, the average number of self-administered injections over the 15 sessions was higher in LEW rats than in F344 rats, as previously shown^9,10^. Our data reinforce the fact that the LEW strain is a morphine-vulnerable phenotype predisposed to higher doses of morphine intake than the F344 strain.

The role of individual differences in the responses to drugs of abuse, and thus predisposition to addiction is not new, and it has been widely demonstrated in humans and laboratory animals^4^. An important issue in drug addiction is to understand whether a person is predisposed to addiction. Thus, differences in vulnerability to drug dependence may be associated with differences in the efficiency of neural substrates to translate the drug effects and, in turn, to differences in brain metabolism. In this respect, the development of PET scanners for laboratory animals enabling the in vivo study of the neurotoxic effects of drugs in a non-invasive follow-up provides an alternative way to explore the underlying mechanisms of morphine-induced neurotoxicity. In the present study, [^18^F]-FDG was used as a marker of cerebral glucose consumption, which indicates neuronal activity^49^. Our [^18^F]-FDG data showed that the LEW animals exhibited increased glucose metabolism in cortical areas, including the somatosensory and the entorhinal cortices and the hypothalamus compared with the F344 animals. These areas are of great importance in brain reward circuits^50^, and may account for the differences between the studied strains. Specifically, the orexigenic neurons located in the lateral hypothalamus^51^ plays an important role in morphine-induced reward, withdrawal, and synaptic plasticity^50^. Therefore, the number of LH orexin neurons has been proposed as a potent predictor of addiction vulnerability^52^. The entorhinal cortex is the main input and output structure of the hippocampus, and the hippocampus is critical for the memory processes of drug-seeking and drug-conditioned stimuli^53^. Thus, in studies with natural reinforces such as food, there are differences in learning between LEW and F344 rats, with LEW rats learning faster than F344 rats^54^. Sensory system information plays an important role in responses related to drug addiction, and, specifically, the somatosensory system is required for the positive reward property of drugs^55^. In this sense, the higher level of glucose metabolism in this area in the LEW rats might indicate differences in sensory information between the strains.

In general, addiction involves pathological learning in the neural processes related to the reward system and, therefore, underlies long-term associative memory deficits^56^. Here, we demonstrated differences in glucose metabolism in the entorhinal cortex between LEW and F344 rats. This area is closely related to learning and memory and connects the hippocampal neocortex and the hypothalamus, which is the main source of afferents to the hippocampus^57^. Research has shown genetic differences between both strains in synaptic plasticity in the hippocampus^58,59^, which are translated into spatial learning and memory deficits. Thus, F344 animals are less effective at performing some behavioral tasks, such as the radial arm maze test and the Morris maze test^60^, in that they are more liable to errors and take longer to learn the task^61^. The increased metabolism in this area in the LEW strain, together with the fact that more morphine was self-administered than the F344 strain, might be related to the higher learning capacity shown in the LEW strain.

We also found higher metabolism levels in the hypothalamus (including the mammillary bodies, the VTA, and the medial forebrain bundle) in the LEW animals than in F344 animals. Of note, the mammillary bodies are also involved (with anterior and dorsomedial nuclei of the thalamus) in recognition memory^62^. Together with the increased metabolism in the cortical area, these results represents a plausible explanation for the greater learning capacity of the LEW strain. In addition, the hypothalamus is part of the hypothalamic–pituitary–adrenal (HPA) axis, and differences in HPA axis reactivity between LEW and F344 strains have been reported^47^. Therefore, the increased glucose metabolism in the hypothalamus in the LEW strain would support this alteration in the HPA axis, which is consistent with the alteration of the reward and motivational processes reported for these strains^63^.

In contrast, glucose metabolism was lower in the brainstem and PAG in the LEW strain than in the F344 strain. The brainstem controls basic vital functions, such as heart rate, breathing, and sleeping, but it is also involved in emotional responses and episodes of distress. In this respect, an increased state of anxiety in LEW rats has been associated with alteration of the HPA axis^63^. Furthermore, while the PAG is a key area in acute and chronic pain processing, it is also involved in mediating fear-evoked behavior, which is in turn related to anxiety and depression^64^. Taken together, these results could also account for the differences in HPA axis reactivity between the strains.

Regarding the morphine self-administration study, during acquisition, morphine-F344 animals showed increased glucose metabolism in cortical areas, with more changes in the left hemisphere than in the right, and decreased FDG uptake in the motor and piriform cortices than saline-F344 animals. These changes were mainly maintained during drug withdrawal. However, the metabolism pattern of the morphine-LEW animals was completely different when compared to saline-LEW animals, with decreases in the somatosensorial cortex, thalamus, and cingulate cortex that were maintained and extended during withdrawal. The different brain metabolic patterns observed after the MSA study between these rat strains indicate differences in the efficiency of neural substrates to translate the drug effects and, in turn, possible differences in vulnerability to morphine abuse.

Few in vivo imaging studies have evaluated the effect of morphine or other opioids on glucose metabolism using PET or single photon emission computed tomography (SPECT) in humans, probably because of the radioactive nature of these techniques, being most of them from the 1990s and beginning of this century. London and coworkers showed that acute administration of morphine in humans reduces glucose uptake in the brain by 10% on average^32^, with more changes in the left hemisphere than in the right hemisphere. In our study, repeated exposure to morphine induced different patterns of brain changes depending on the rat strain. During acquisition phase, morphine resulted in reduced glucose metabolism in cortical areas in both strains. This pattern of cortical reduction increased after withdrawal in the LEW animals but not in the F344 animals. Repeated exposure to morphine is usually accompanied by the development of tolerance and dependence. In addition, although the exact mechanisms underlying these phenomena are not yet fully understood, they are known to be associated with drug-induced neuroadaptations^56^. In this respect, nuclear magnetic resonance spectroscopy studies demonstrated that acute administration of morphine produces a significant decrease in glycine and glutamate levels that were dramatically increased or overcompensated, when naloxone was used to precipitate withdrawal^65^. Thus, in our F344 animals, chronic morphine may have triggered some compensatory mechanisms in order to normalize glucose metabolism, which were not triggered in the LEW animals.

During drug withdrawal in the LEW strain, glucose metabolism decreased dramatically in brain regions associated with reward and drug dependence, such as the hippocampus, thalamus, caudate-putamen, and cingulate cortex; while glucose metabolism changes in the F344 strain were modest compared to the LEW strain. In humans, neurobiological abnormalities in the regional cerebral metabolic rate for glucose were found in chronic opiate users several years after methadone detoxification^66^. This widespread pattern of abnormal cortical activity involved the anterior cingulate cortex, left mid-cingulate cortex, left insula, and right superior frontal cortex^66^, similar to some of our cortical changes. In addition, SPECT studies have shown perfusion deficits during heroin withdrawal in several brain areas, including the temporal lobe^67^ and the frontal, parietal, and temporal areas in a chronic opioid user after one week of interrupted administration^68^. In our study, 15 days of morphine abstinence induced a pattern of abnormal brain activity in the LEW animals similar to that found in humans. However, this abnormal metabolic pattern was not found in the F344 animals, suggesting that the genetic background (and other factors) makes one individual more susceptible to developing morphine addiction than another. Furthermore, the LEW strain showed increased metabolism in the piriform cortex. This structure is an olfactory region, and its involvement in the behavioral effects of drugs is limited. Thus, the increased expression of the activity marker c-Fos in the piriform cortex has been associated with cocaine-induced conditioned place preference^69^ and has also been associated with relapse in opioid seeking after food choice–induced voluntary abstinence^70^. Therefore, the increased metabolism in the piriform cortex in the LEW strain could respond to morphine seeking after the withdrawal period and could explain in part the implication of the piriform cortex in morphine addiction and dependence.

Nonetheless, our study had several limitations. First, we only evaluated males. The effect of morphine on females could be different, so further studies would be advisable to determine how the gender may influence on the effect of morphine in brain metabolism. Second, we have not corrected for multiple comparisons since individual analysis methods (SPM) provide some correction, being a common practice in exploratory works^71^. In addition, Bonferroni correction assumes independence of the voxels, which is not true in brain imaging studies and would underestimate the real effects.

In conclusion, we found significant brain metabolic differences between LEW and F344 strains in brain regions associated with reward and drug dependence. In addition, the different brain metabolic patterns observed after the MSA study between these rat strains indicate differences in the efficiency of neural substrates to translate the drug effects, which could explain the differences in predisposition to morphine abuse between one individual and another. These findings have important implications for the use of these rat strains in translational morphine and opiate research.
